# Giant Nonlinear Optical Absorption of Freestanding Graphene Oxide Films for Femtosecond Pulse Compression

**DOI:** 10.1021/acsami.5c10300

**Published:** 2025-07-18

**Authors:** Rowoon Park, Sang-Hyuk Park, Minwoo Kim, Minju Kim, Seungho Park, Young Woo Kwon, Songyi Lee, Kwangseuk Kyhm, Suck Won Hong, Robert A. Taylor

**Affiliations:** † Department of Optics and Mechatronics Engineering, Department of Cogno-Mechatronics Engineering, and College of Nanoscience and Nanotechnology, 34996 Pusan National University, Busan 46241, Republic of Korea; ‡ Engineering Research Center for Color-Modulated Extra-Sensory Perception Technology, Pusan National University, Busan 46241, Republic of Korea; § Department of Physics, 6396 University of Oxford, Oxford OX1 3PU, U.K.; ∥ Department of Chemistry, Pukyong National University, Busan 48513, Republic of Korea

**Keywords:** graphene oxide, freestanding film, femtosecond laser, nonlinear optics, pulse compression

## Abstract

We have successfully produced an ultrathin freely suspended GO film, which is a biomimetic structure inspired by the transparent dragonfly wing structure. Based on a colloidal self-assembly process over a large area, solvent evaporation was applied within a limited opening geometry. The free-standing GO film shows a significant enhancement of the nonlinear optical absorption, where saturable absorption and photoinduced absorption were observed at dramatically decreased excitation fluence compared with other work on GO films dispersed on substrates. Surprisingly, we also found that free-standing GO films are beneficial for compressing femtosecond pulses around 800 nm. Using a frequency-resolved optical gating as well as an open aperture Z-scan method, the origin was found to be associated with two effects. While the pulse shortening results from saturable absorption, the chirp effect is also suppressed due to the presence of an inflection point around 800 nm in the refractive index spectrum of free-standing GO film.

## Graphical abstract

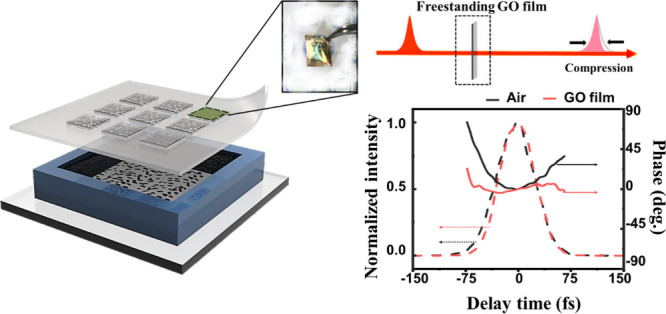

## Introduction

The exploration of two-dimensional (2D) materials began with the groundbreaking discovery of single-layered graphene, whose unexpected and exotic properties at atomic thickness have attracted widespread interest in various research fields leading to numerous practical engineering applications.^1–3^ The unique properties of graphene arise from its sp^2^-hybridized carbon atoms arranged in a honeycomb lattice, exhibiting superior electrical mobility, extraordinary mechanical strength, and high thermal conductivity.^4,5^ Most of the novel electronic and optical properties of graphene are explained in terms of massless Dirac Fermions with linear energy dispersion in its gapless band structure at the Dirac point.^6^ This linear dispersion gives rise to ultrafast carriers with ballistic transport even at room temperature.^7^ Under strong excitation, the gapless energy dispersion of graphene exhibits broad saturable absorption (SA) in photon-induced electronic transitions. This can be attributed primarily to the Pauli blocking effect, where the filled states in the conduction band prevent further electronic excitation.^8^ This phenomenon leads to broadband SA. Therefore, graphene functions as a highly effective optical limiter, and these nonlinear optical characteristics have also been exploited to generate broadband tunable femtosecond laser pulses for passive mode-locked lasers.^9,10^ Furthermore, the ultrafast carrier dynamics in graphene results in subpicosecond recombination time and high damage threshold under intense laser irradiation. This characteristic is also useful for ultrafast photonics and pulse shaping applications.^11^ Recent advances have also demonstrated that the optical properties of graphene can be tuned through chemical doping or strain engineering, whereby its potential application can be expanded to optoelectronics, tunable photodetectors, modulators, and terahertz sources.^12^ Nevertheless, the broad absorption of graphene over a wide range of wavelengths needs a spectral modification to design devices with a selective narrowband response for optical modulators and detectors.

Graphene oxide (GO), a member of the graphene family of materials, is a derivative of two-dimensional graphene decorated with oxygen-containing functional groups (OFGs).^13^ While graphene consists of *s* *p* ^2^-hybridized carbon orbitals with σ bonds with the remaining p orbitals perpendicular to the plane for π bonds, some of the carbon atoms in GO are sp^3^-hybridized with OFGs, such as epoxides, hydroxyls, and carboxyls, which are attached to the carbon network.^14^ As a result, GO has a heterogeneous structure with an opened bandgap, where the nanographene domains of *sp* ^2^-hybridized orbitals are separated by functionalized and oxygenated *sp* ^3^-carbon network, and its opened bandgap can be tuned by varying the size and fraction of the *sp* ^2^ domain.

In the context of nonlinear optical (NLO) responses,^15^ carbon-based materials have been considered as broadband optical limiters such as carbon black suspensions (CBS) and carbon nanotubes (CNTs).^16^ Pristine graphene materials have also received significant attention for their large NLO properties. However, they suffer from structural issues that make bandgap tuning difficult, often requiring sophisticated microprocessing to fabricate devices. On the other hand, GO offers several advantages that make it a promising candidate for various applications. It can be synthesized from inexpensive graphite using cost-effective chemical methods, and its high hydrophilicity allows it to form stable aqueous colloids.^17^ This facilitates its assembly into macroscopic structures via simple and inexpensive solution processes. Furthermore, the high solubility of GO in various solvents and the ease of preparing liquid dispersions enable it to form stable film structures,^18^ making large-scale fabrication possible and overcoming practical limitations.

To date, Z-scan and transient absorption techniques have been employed in transmission geometries to study the third-order NLO properties of GO.^19^ However, pulse propagation characteristics have never been considered as substrate effects were unavoidable. For example, even transparent materials results in chirp effect.^20^ Therefore, a freely suspended GO film is necessary to compare its intrinsic NLO properties against the chirp effect, and this consideration is crucial for practical applications of GO film-based photonics. As a member of the 2D material family, GO exhibits significant potential in the field of LNO research due to its ease of preparation and tunable physicochemical properties.^21^ GO is also valuable for the emerging technologies of flexible photonics and optoelectronic devices.^22^ However, it remains crucial to address the challenges associated with large-area film fabrication for constructing optical modules using planar components of layer-transferred freestanding GO films. The ability to tailor the unique properties of GO films to meet the requirements of diverse devices could add substantial value by enabling the development of versatile, multifunctional optical elements that can be integrated into complex systems.^23^ Recent advancements have made it possible to control large-scale stacked GO nanofilm structures, combining standard photolithography and lift-off processes.^24^ This allows for precise positioning when coating GO films on integrated photonic devices.

Herein, we report the successful fabrication and extensive optical characterization of ultrathin freestanding GO films for the observation of unique pulse shortening effects, excluding unwanted optical effects that may arise from the substrate. To overcome the challenges that hinder optical measurements, we employed a solvent evaporative colloidal self-assembly process over a perforated surface area to modulate the tunable thickness GO films. Combining this unique geometry and a tuned GO concentration, we produced freestanding GO film arrays without a precise transfer process. This enables us to study the transient response of GO film to Ti:sapphire femtosecond laser pulses using frequency-resolved optical gating (FROG) and Z-scan methods, whereby the temporal evolution of femtosecond pulses was analyzed and the SA and TPA were quantified in terms of the characteristic nonlinear optical coefficient for excitation fluence. Surprisingly our results show that freestanding GO films give rise to pulse compression by the SA with a suppressed chirp effect due to the presence of an inflection point in the refractive index spectrum. These findings will open important applications of GO in ultrafast optics and photonics.

## Methods and Materials

### Production of Freestanding GO Thin Films

Freestanding GO thin films were fabricated via the self-assembly process of individual GO sheets in the confined geometry consisted of PDMS well and perforated PET guide films. To produce PDMS well, PDMS prepolymer (Sylgard 184, Dow Corning) was mixed with a curing agent in a 10:1 weight ratio and poured into the square dish. After degassing and thermal curing at 80 °C for 2 h, the cured PDMS film, with a thickness of 3 mm, was peeled off from the square dish. PDMS film with the size of 22 mm (width) × 22 mm (length) was then transferred onto a glass substrate and cut into a square hole to an interior size of 20 mm (width) × 20 mm (length). Then, a PET guide film with a thickness of 25 μm was perforated with a size of 10 mm (width) × 15 mm (length) and covered after filling with a GO solution ranging from 0.5–2.0 mg mL^–1^ onto the PDMS well. After thermal evaporation at 100 °C for 10 min, the convex GO droplets gradually evaporated and formed a concave GO/water layer on the perforated edge of the PET guide film. After that, the PET guide film containing the GO/water layer was carefully peeled off from the PDMS well and dried in ambient conditions.

### Characterization

The morphology of the freestanding GO film was observed using optical microscopy (OM, Olympus BX50, Tokyo, Japan) and scanning electron microscopy (SEM, SUPRA40VP, Zeiss, Oberkochen, Germany). The surface topology of the freestanding GO film was analyzed using atomic force microscopy (AFM, Park Systems, Suwon, Korea). Raman spectroscopy, with a 532 nm laser excitation (UniNanoTech Co., Yong-In, Korea), was employed to characterize the freestanding GO film. The surface chemical states of freestanding GO thin films were investigated using XPS (Kratos Analytical, AXIS SUPRA, Japan). The optical transmittance of the freestanding GO film, prepared at various GO concentrations, was measured in the wavelength range of 400–800 nm using UV–vis spectroscopy.

### Numerical Simulations

The temperature distribution and flow velocity of the GO droplet within the confined geometry consisting of perforated PET guide film and PDMS well were simulated using COMSOL Multiphysics (Version 5.4). The Heat Transfer in Fluids and Laminar Flow modules in software were employed to model the thermal and fluid dynamics behavior as the system was heated from 25 to 100 °C. The geometry of the PDMS well and perforated PET film was constructed within COMSOL’s modeling environment and discretized using a tetrahedral mesh. The entire system, including the GO solution, PDMS well, and PET film, was initially set to a uniform temperature of 25 °C. The temperature of the system was increased uniformly from 25 to 100 °C over a specified time period, simulating a controlled heating process. The external environment was assumed to be thermally insulated to ensure that heat transfer occurred primarily within the system. The GO solution was initially at rest (i.e., zero velocity). As the temperature increased, natural convection was modeled to drive the flow within the well. The perforations in the PET guide film allowed for fluid movement, with a no-slip boundary condition applied to the walls of the PDMS well and the surface of the PET film.

The temperature distribution and flow velocity within the GO solution were governed by the coupled Navier–Stokes and heat transfer equations.

The Navier–Stokes equation for incompressible flow was used to model the fluid dynamics:^25^

1 $$\rho \left(\partial u/\partial t+u\cdot \nabla u\right)=-\nabla p+\mu \nabla^{2}+F$$

where ρ is the fluid density, *u* is the velocity vector, *p* is the pressure, and μ is the dynamic viscosity.

Heat transfer within the GO solution was modeled using the heat equation:^26^

2 $$\rho C_{p}\left(\partial u/\partial T+u\cdot \nabla T\right)=k\nabla^{2}T+Q$$

where *C*_*p*_ is the specific heat capacity, *k* is the thermal conductivity, *T* is the temperature, and *Q* represents any internal heat sources.

The simulation was conducted in a transient mode to capture the time-dependent temperature distribution and flow velocity as the temperature increased from 25 to 100 °C. The heating rate was controlled to simulate realistic experimental conditions, ensuring that the temperature gradient and resulting fluid flow within the PDMS well could be accurately analyzed.

## Experimental Section

### Z-Scan Measurement

The nonlinear absorption property of the freestanding GO film was characterized by the Z-scan technique^27^ as illustrated in Figure S8a. 100 fs pulsed laser (800 nm with 80 MHz repetition rate) was focused to sample by an objective lens (focal length: 500 mm), generating a beam waist of ∼5 μm at the focal point.^28^ A neutral density filter was employed to vary input laser fluence continuously. The freestanding GO film was mounted on the custom-made 3D-printed gasket and then held by commercial optical components. Using a motorized linear translation stage, the position of sample was scanned across the focal region along the *z*-axis. Consequently, the transmitted beam was collected by detector as a function of *z*.

### Frequency-Resolved Optical Gating (FROG)

Figure S8b shows FROG autocorrelation setup, whereby the temporal pulse profile can be analyzed in terms of intensity and phase.^29^ Initial laser pulses (tuned at 800 nm) become split by a 50/50 beam splitter, and one of the paths is directed to movable retroreflector, whereby time delay to the other path is induced. The pair of delayed pulses is focused to a beta barium borate (BBO) crystal for second harmonic generation, and the frequency-doubled spectrum of the laser pulses are measured by CCD camera attached to monochromator. Given the SHG map for wavelength and delay time, which is called spectrogram, the temporal profile of intensity and phase can be extracted using the FROG analysis. Compared with the spectrogram of a reference pulse in air, the transmitted pulse with freely standing GO film gives rise to a different spectrogram.

## Results and Discussion

### Fabrication of Freestanding GO Membrane Arrays: Bioinspired Design

Inspired by the unique structures of transparent dragonfly wings, freestanding GO films were prepared as a biomimetic membrane. From the perspective of structural design, dragonfly wings consist of a venation network and wing membrane (Figure 1a). The transparent thin membranous areas are composed of an exocuticle and a thin layer of mesocuticle, arranged in a sandwich structure and positioned between a network of longitudinal and transverse veins.^30^ The composition of the mesocuticles may vary significantly between the wing membrane adjacent to the veins and the membrane located in the center of a wing membrane area or cell.^31^ Depending on their size and overall connection to surrounding veins and the wing membrane, these membrane areas experience varying degrees of bending, buckling, and torsional deformations.^32^ Motivated by this vein and membrane structure of dragonfly wings, our experimental approach employed a perforated polymeric guide film to serve as a vein skeleton, with a freestanding GO film functioning as a membrane matrix. As schematically illustrated in Figure 1b, a simple process was delicately used to produce a freestanding GO film by assembling colloidal GO sheets dispersed in water by spontaneous solvent evaporation.^33,34^ Our developed strategy mainly centered on the use of the evaporative self-assembly of GO sheets into a stacked and suspended film supported by a perforated polymer thin film. The first step involves confinement of the GO solution in a conformally fixed well (polydimethylsiloxane, PDMS) on a glass substrate (Figure 1b (i)). The height of the PDMS well was typical set as ∼3 mm and fully filled with concentrated GO solutions (*c* = 0.5, 1.0, 1.5, and 2.0 mg mL^–1^), which was one of the main parameters necessary to tune the final thickness level of the freestanding GO films.

**Figure 1:**
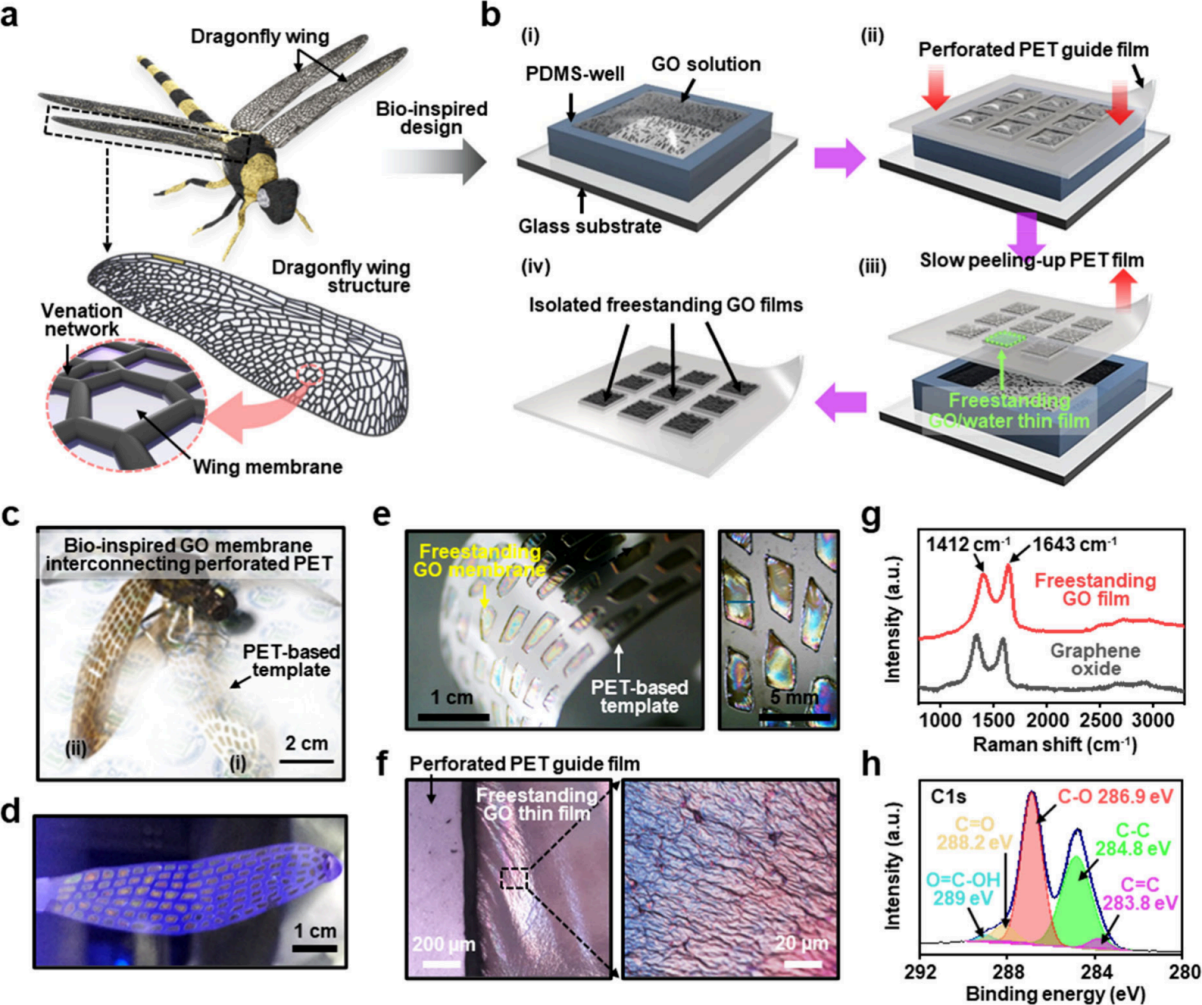
a) The bioinspired design of dragonfly membranous wings motivated the construction of freestanding GO thin films; the wing structure in dragonflies is mainly composed of veins and membranes. b) Schematic illustration for fabricating isolated freestanding GO membrane in-between the perforated PET guide film. c) The Biomimicking of dragonfly wings based on freestanding GO thin films interconnected in the perforated PET template; forewings (i) and hindwings (ii) were prepared by GO solutions with different concentrations of 1 mg mL^–1^ and 2 mg mL^–1^, respectively. d) A membrane of the dragonfly wing produced by the self-assembly of GO sheets; the inset image shows the freestanding GO membrane fabricated by mixing GO sheets and quantum dots. e) A magnified digital image of the freestanding GO membrane formed in the perforated PET film. f) An optical microscope image showing the freestanding GO membrane with an uneven surface; the magnified image shows the structural color due to thin film interference. g) A Raman spectrum of a freestanding GO film and drop-cast GO film. h) C 1s XPS spectra of the freestanding GO thin films.

In a similar manner to the formation process of dragonfly wings, where the distribution of cuticles and surrounding blood vessels make up the wing structure, the freestanding GO films can be greatly influenced by the GO concentration and the domain size of the perforated polyethylene terephthalate (PET) film. In our experimental procedure, a transparent PET guide film was placed carefully on the PDMS well edge surface to cover the fully filled GO solution. At this moment, spontaneous trapping of the solution occurs due to capillary forces. The key technical point of this experiment is to provide a balanced surface tension environment for the GO solution at the local fixation site by wetting the GO solution in a defined area (i.e., a millimeter-sized rectangular cavity) using the perforated PET guide film (Figure 1b (ii)). Under these limited geometric conditions, after the water solvent gradually evaporates completely, allowing the PET guide film to be peeled off slowly to avoid physical destruction of the freestanding GO film formed on it. Through this method, we successfully generated large-scale separately isolated freestanding GO films in the perforated area of the PET guide film. In detail, as presented in Figure S1, each sequential side-view schematic illustrates the self-assembly steps as the water evaporates. When the PET guide film was placed smoothly on the PDMS well, the GO solution protruded only slightly through the uncovered areas, enabling a surface tension-driven trapped GO solution to be precisely situated. Then, the homogeneously distributed GO sheets in water could be assembled in a stacked-configuration by the volatile solvent evaporation, along with the liquid–air interface. In fact, this entrapment of the liquid film is a commonly observed phenomenon in daily life when children use bubble play by passing liquid soap through the mesh-type net.^35^ We utilize the GO solution in a similar manner to produce specific conditions that leave behind the GO-sheet solutes only on the perforated PET film areas by water evaporation. As shown in Figure S1 (iii), the water in the GO solution continuously evaporated from the surface of the solid PET film. This process led to a gradual decrease in the concentration of the GO solution from the upper to the lower area within the well, allowing the spontaneous progression of the accumulation of GO layers in the isolated perforated areas.^36^ As a result, the GO deposition was horizontally fixed on the PET guide film. A well-known viscous liquid crystallinity of GO was facilitated in the freestanding GO film with sufficient mechanical robustness by capillary forces via water evaporation and van der Waals interactions within the individual GO sheets.^37^ In other words, at the initial stage of freestanding structure formation, the loosely packed GO films allow partial water vapor permeation due to structural imperfections. This process gradually enhances the structural integrity as the GO films become tightly anchored and fully interconnected on the PET guide film area.^38^ Consequently, in the final stage of water evaporation from the GO solution, the freestanding GO films undergo gradual densification, ultimately completing the process as illustrated in Figure S1 (iv). Therefore, our strategy is not limited in scalability because the localized entrapment of the GO solution can be occurred with the help of the PET guide film.

As a biomimetic approach was proposed earlier, Figure 1c presents a digital image demonstrating the structural similarity between dragonfly wings and freestanding GO film on a perforated PET. The dragonfly wing model consists of two pairs of wings: forewings (camel-colored) and hindwings (dark brown), fabricated with GO concentrations of 1.0 and 2.0 mg mL^–1^, respectively, to illustrate the ease of thickness tuning within the provided PET guide film. Despite the random distribution of perforated domain sizes, a relatively homogeneous GO film formation was achieved in a freestanding form (Figure 1d). To verify this further (inset image in Figure 1d), the freestanding GO films were produced from a mixed solution of GO sheets and dye (i.e., quantum dots), which emit different light (red) compared to areas of the PET-guided film (blue), confirming the formation of a freestanding GO film that is not in contact with the substrate. Additionally, such GO membranes with PET as a support layer in the shape of dragonfly wings, formed through self-assembly of GO sheets, exhibited high transmittance, as shown in Figure 1e. An enlarged digital image demonstrates freestanding GO films interconnected with PET guide film in a bent state, confirming that the stacked GO films are tightly anchored without damage and retain sufficient mechanical properties. The enlarged digital image in the right panel shows the independently organized freestanding GO film domains that exhibit structural colors associated with thin-film interference, depending on the angle of light incidence.^39^ Generally, structural colors are sensitive to surface morphology and film thickness, which are characteristic of light interference.^40^ However, due to micro/nanoscale scattering on the surface of the freestanding GO film,^41^ structural color reflections appear only on the GO membrane surface, in contrast to the PET guide film surface. The magnified optical micrographs in Figure 1f show a relatively homogeneous surface composition for the freestanding GO film, demonstrating strong van der Waals forces within the perforated area and clarifying robust mechanical properties in the bent state; the magnified image in the right panel shows a local surface structure, revealing the structural color.

To determine the chemical composition of the self-assembled, freestanding GO film surface, the sample surface was characterized using Raman spectroscopy and X-ray photoelectron spectroscopy (XPS). As presented in Figure 1g, the collected Raman spectrum exhibits typical spectral features of graphene-based materials, with two major bands assigned to the D band (1412 cm^–1^) and G band (1643 cm^–1^). Briefly, the D band is attributed to the reduction in size of the sp^2^-bonded carbon lattice due to sp^3^ structural defects, vacancies, and distortions during oxidation.^42^ The G band corresponds to vibrations of carbon atoms in the sp^2^ region and indicates the crystal structure state.^43^ The *I*_D_/*I*_G_ peak intensity ratio was found to be ∼0.94, indicating the density of defects in the GO sheet and, simultaneously, the proper introduction of oxygen functional groups. The surface chemical structure analysis in Figures 1h and S2 shows the C 1s and O 1s spectra of the freestanding GO film. The C 1s XPS spectrum is characterized by C=C/C–C (sp^2^ or sp^3^ carbon, ∼284.8 eV), C–O (hydroxyl or epoxy, ∼286.9 eV), C=O (carboxyl, ∼288.2 eV), and O=C–O (carboxyl, ∼289 eV) emission. Additionally, the O 1s spectrum indicates that the GO film formed through strong oxidation on graphite contains various oxygen functional groups (Figure S2). The oxygenated carbon peaks are distributed at binding energies of ∼532.4 and ∼533 eV, corresponding to C=O/O–C=O and C–OH/C–O–C bonds, respectively. These comprehensive measurements confirmed that the surface chemical structure of GO maintains its integrity in the stacked array, even during the evaporative self-assembly process used to fabricate the freestanding GO thin films.

### Large-Scale Freestanding GO Thin Films to Prepare Optical Modules

Based on the results exemplified by the biomimetic approach, the technique introduced in this study was extended to fabricating optical modules using freestanding GO films. As illustrated in Figure 2a, the sequential process to fabricate large-scale freestanding GO films by increasing the perforation area of the PET guide film is demonstrated. Through this approach, highly organized stacked structures of GO sheets were formed along the air/liquid interface of the GO solution filled in the PDMS wells through collective interactions such as hydrogen bonding, van der Waals forces, and electrostatic interactions.^44^ In contrast to our previous experiments, the fabrication process of large-area freestanding GO films employed slightly elevated temperature conditions to accelerate the evaporation rate at the GO solution/air interface. After the sufficient evaporative process, the PET film, initially attached to the PDMS well, was slowly lifted to separate the layered GO film (i.e., gel-like film state) formed at the upper layer where partial evaporation occurred, thereby maximizing the self-assembly of GO colloids (i.e., multistacked configuration) connected to the perforated PET boundaries. As previously reported, for colloidal GO sheets dispersed in water, surface tension forces drive the GO meniscus along the tangent to the water–air interface during solvent evaporation.^45^ From a microscale perspective, the GO meniscus gradually transitions from a convex to a concave shape over time during solvent evaporation when confined by the perforated PET guide film. Meanwhile, the edges of the perforated PET boundaries were tethered with inwardly concave GO films induced by capillary forces, allowing the guide film to be carefully separated from the PDMS well (depicted in the second panel of Figure 2a). Subsequently, the concave GO/water films trapped in the perforated area of the PET film could be dried slowly, resulting in the formation of freestanding GO films with unique surface structures and transparent properties, analogous to the dragonfly wing-inspired GO films (Figure 1e–f). Furthermore, inspired by the vascular structure of dragonfly wings, the supporting guide film (i.e., perforated PET film) that anchors the GO film offers the advantage of being independent of the perforation shape during the fabrication of a free-standing film via evaporation-induced assembly of GO sheets at the air/liquid interface (Figure S3).

**Figure 2:**
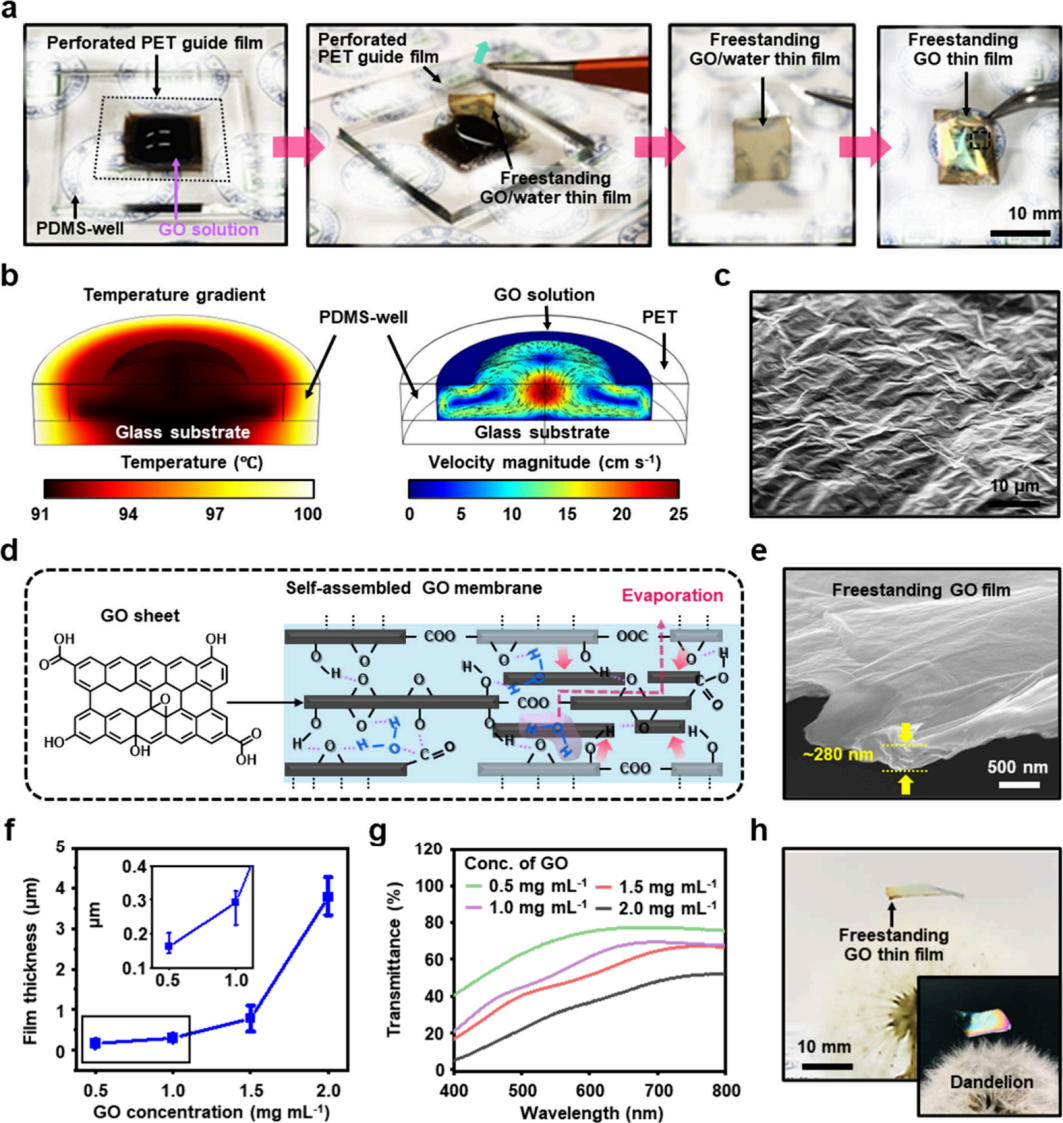
a) A series of digital images of the manufacturing process for large scale freestanding GO thin film with square shape. b) Simulated temperature distribution (left) and velocity magnitude (right) while the water evaporates from experimental geometry consist of the PDMS well and perforated PET guide film. c) An SEM image observed on the surface of the freestanding GO membrane near the center. d) The self-assembly behavior of GO sheets in a GO-water membrane entrapped by capillary force between PET edges; the water molecules interlink GO sheets situated in different planes through hydrogen bonding. e) Cross-sectional view SEM image of a freestanding GO membrane. f) Thickness level of freestanding GO thin films as a function of the applied GO solution concentration. g) Optical transmittance of free-standing GO thin films prepared according to GO concentration; each solid line corresponds to a concentration of 0.5 (green), 1.0 (purple), 1.5 (red), and 2.0 mg mL^–1^ (gray). h) Side-view images of the freestanding GO thin films placed on the dandelion seed.

As detailed above, global heating of the PDMS well/glass substrate was a crucial factor in the experimental design during the drying process. To investigate the formation process of the freestanding GO films, we computed the heat flow of the GO solution by applying the physical parameters required for the formation of the freestanding GO film structure. Figure 2b illustrates the simulated temperature and velocity (i.e., flow rate) within the GO solution trapped in the PDMS well, providing reasonable information and a general view of the deposition process of GO colloids. This simulation allowed us to postulate the formation mechanism of a connected tether structure to the perforated PET guide film as the solvent evaporates from the GO solution in the well. Based on previously reported physical parameters, we estimated the temperature distribution between the interior and the edge of the GO solution in the local area confined by the perforated PET guide films. The closed chamber temperature was maintained at 100 °C under atmospheric pressure to accelerate the evaporation rate of the GO solution, which was initially filled in the PDMS well in a convex shape with an angle θ of 70° (Figure S4). Under these conditions, the inward and outward convective flow within the GO solution, induced by heat transfer upon solvent evaporation, generates thermocapillary forces. These forces result from the surface tension gradient caused by temperature changes (from 100 to 91 °C) at the GO solution surface due to contact with the surrounding air. The convective flow of the GO solution inside the well, driven by external heating, significantly influences the final film structure. This effect can be directly compared with the results of freestanding GO films damaged in some areas when the experiment was performed without a heating process (i.e., natural evaporation at ∼25 °C). Notably, the relatively slow flow of the GO solution observed around the perforated PET guide film (∼5 cm s^–1^) greatly facilitates stable film formation, while the relatively faster flow (∼10–13 cm s^–1^) in the central region keeps the GO colloids suspended, enabling continuous deposition of GO at the air/liquid interface. These hypotheses and experimental observations clearly confirm the formation of large-area freestanding GO films. Figure 2c presents an SEM image of the surface morphology of the resulting GO thin film, which is similar to the optically measured images (Figure 1f). Specifically, the nanoscale rippled surface of the freestanding GO film appears to result from the process in which individual GO sheets are stacked along the initially formed GO film by convection in the water layer during the drying process.^46,47^ Furthermore, regardless of the GO concentration, the stacking of individual GO sheets exhibits a rippled surface morphology, emphasizing that heat transfer inside the PDMS well is a critical parameter in the GO film formation process (Figure S5).

Figure 2d presents a schematic representation of the evaporative self-assembly mechanism at the air/liquid interface. During this process, the assembly of 2D GO colloids occurs as solvent evaporation drives the accumulation of GO sheets at the molecular interface.^33^ As the solvent evaporates, GO sheets initially dispersed in the liquid phase are drawn together by capillary forces, and the functional groups on GO, including hydroxyl, carboxyl, and epoxy groups, simultaneously interact through hydrogen bonding and van der Waals forces, promoting the alignment and stacking of the GO sheets into a densely packed structure. This self-assembly process, particularly effective at the air/liquid interface, is conducive to the formation of continuous, freestanding GO films. As noted, although the process is governed by several parameters such as GO concentration, evaporation rate, and the physical-chemical properties of the liquid medium,^48^ the chemical interactions between GO functional groups also contribute to the structural stability and integrity of the assembled films, significantly affecting the degree of overlap, packing density, and uniformity of the final freestanding GO film.^49,50^ In Figure 2e, the large-area freestanding GO assembly resulted in a film thickness of ∼ 280 nm, as measured by side-view SEM image, revealing a distinct surface morphology for the stacked GO films (Figure S6). The variation in film thickness with respect to GO concentration (i.e., 0.5, 1.0, 1.5, and 2.0 mg mL^–1^) was confirmed to range from the nano- to microscale through SEM measurements, as summarized in Figure 2f. The freestanding GO films gradually increased in thickness with increasing GO concentration range, which serves as a key parameter for modulating the thickness of these films. Additionally, these results were also confirmed by investigating the optical transmittance of the freestanding GO films prepared with varying GO concentrations via UV–vis spectroscopy (Figure 2g). At lower GO concentrations (e.g., 0.5 mg mL^–1^), the films exhibited higher optical transparency, with transmittance exceeding ∼70% at 550 nm. Conversely, films prepared at higher GO concentrations (e.g., 2.0 mg mL^–1^) demonstrated significantly reduced transmittance, dropping to less than ∼ 30.6% at the same wavelength. This observation can be attributed to the increased density and thickness of the freestanding GO films at higher concentrations, leading to greater absorption and scattering of light. The stacked thickness of the GO sheets with the presence of functional groups further gain light interaction effects, contributing to the overall decrease in transmittance^48^ The tunability of the optical properties of the freestanding GO film is particularly important for applications in optoelectronics, where precise control over optical transmittance may be essential.^19^ In this study, we aimed to identify a range of samples that satisfy the requirements for use as optical modules. Notably, to demonstrate the interesting applicability of the prepared freestanding GO films as ultralightweight optical membranes, when placed on dandelion seeds, they exhibited unprecedented light properties that did not affect the seeds (Figures 2h and S7). Collectively, freestanding GO thin films offer unique advantages for ultralightweight optical element with controllable thickness. Furthermore, this freestanding GO thin film can be developed into NLO modules by investigating various types of laser responses, which are affected by the ratio of intrinsically formed structural domains in the GO structure.^50^ Thus, so far, we have prepared a promising solid-state ultrathin material that can easily integrate nonlinear photonic devices.^51^ We will systematically explore the NLO properties of this light-responsive nanomaterial in the following sections.

### Femtosecond Pulse Compression

When a femtosecond pulse passes through a dispersive medium, a change in its pulse duration is unavoidable. The origin of these linear and nonlinear optical effects lies in group velocity dispersion (GVD) and SA. Given a pulse phase in the spectral domain with refractive index spectrum as a function of wavelength *n*(λ), the first order phase term determines the group velocity (GV) *v*_g_ = *c*/*n*_g_, where the group refractive index *n*_g_ = *c*/(*n* – d*n*/d*λ*) is associated with the slope of refractive index spectrum with wavelength (d*n*/d*λ*). On the other hand, GVD is governed by the second order phase term, where the second order derivative of refractive index d^2^ *n*/d*λ* ^2^ is involved. In the case of d*∂* ^2^ *n*/d*λ* ^2^ > 0, the relatively long wavelengths move faster with a large group velocity compared to shorter wavelengths. As a result, the pulse duration becomes broadened with a positive coefficient at the second order phase term in the time domain, which is called positive chirp effect.

As shown in Figure 3a, the effects of GV (d*n*/d*λ*) and GVD (d^2^ *n*/d*λ* ^2^) can be observed by the temporal pulse profile. In the case that the propagating pulse envelope in a medium slows down, a group delay (τ_g_ = *L*/*v*_g_) is induced. With a medium length *L*, the group delay enables us to measure *n*_g_ and d*n*/d*λ* at the central laser wavelength, where the refractive index spectrum *n*(λ) (Figure S9). To measure the temporal pulse profile, we used a cross-correlation technique.

**Figure 3:**
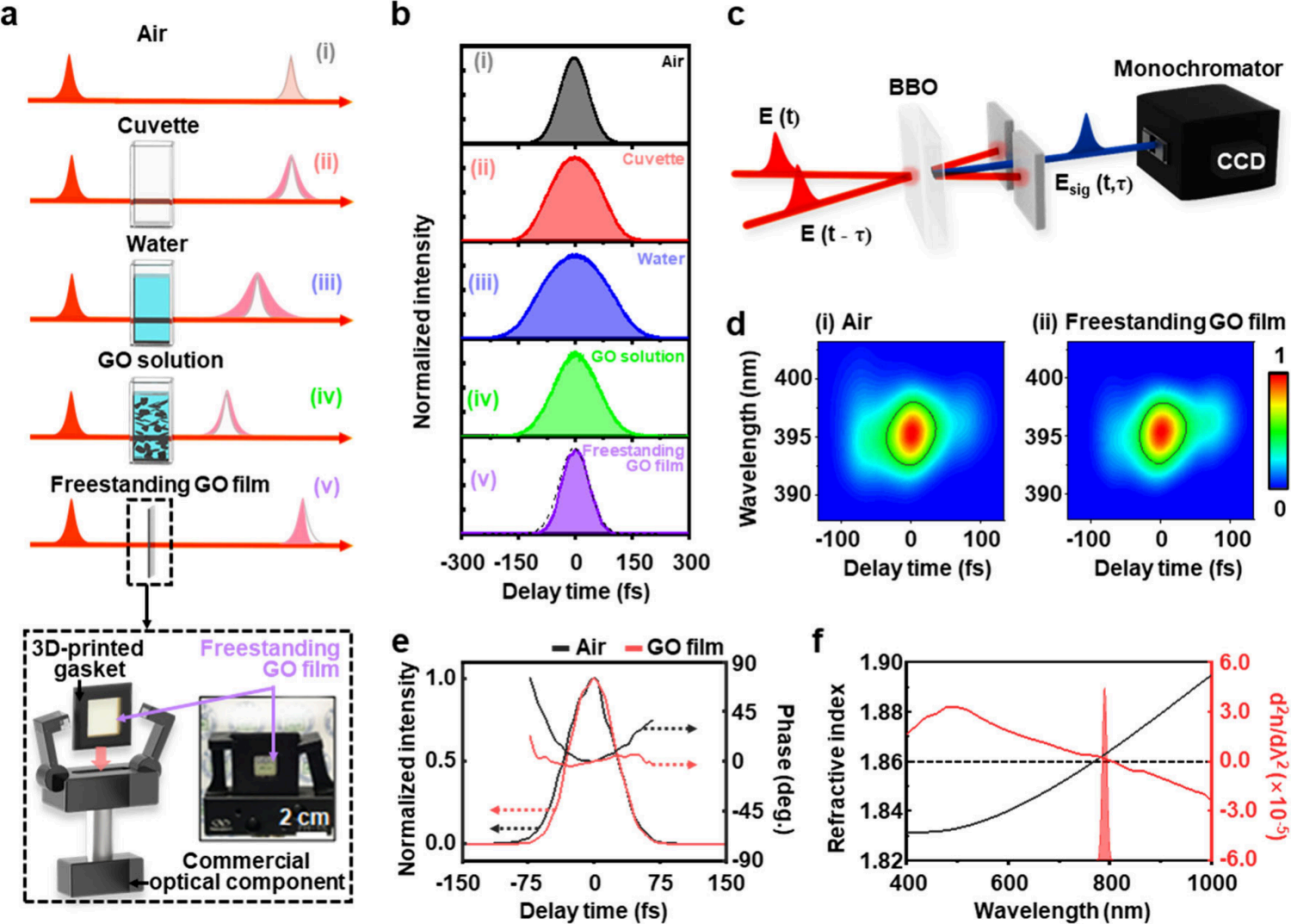
a) Temporal profiles of femtosecond laser pulses measured under different conditions. The pulse in air (i), measured without a cuvette, serves as a reference. Passing through an empty cuvette (ii) and a water-filled cuvette (iii) leads to pulse broadening due to dispersion, whereas a significant pulse compression is observed in the GO solution (1 mg/mL) (iv) and even more so in the free-standing GO film. b) Given a reference pulse in air (i), the temporal intensity profiles (i.e., autocorrelation) of broadened (ii, (iii) and compressed (iv, (v) pulses were compared. c) Schematics shows autocorrelation set up for frequency-resolved optical gating (FROG), where the second-order correlation of a delayed pulse pair is measured in terms of second-harmonic generation (SHG) signal. d) SHG spectrogram for air (i) and free-standing GO (ii), which is SHG intensity map for wavelength and delay time (τ). e) Temporal pulse profiles of intensity and phase for air and free-standing GO film. f) Refractive index spectrum *n*(λ) of a GO film, its second-order derivative spectrum (d^2^ *n*/d^2^λ) shows the presence of an inflection point (d^2^ *n*/d^2^λ=0) near 800 nm.^1^

The sample was positioned in one of the separated beam paths in the interferometer, and the transmitted pulse was combined with a reference pulse in air to generate second harmonic generation (SHG) as a second-order correlation. In Figure 3b, the resultant temporal profiles of laser in various media were compared, which were obtained by the SHG intensity as a function of delay time between the two pulses. Note that the pulse peaks are centered at zero delay time intentionally for easy comparison of the pulse durations although the original data of each pulse shows a group delay (Figure S11).

To establish a clear reference for the input pulse duration, we first measured the autocorrelation of the laser pulse in free-space (without sample), hereafter referred to as ‘air’, yielding a pulse width of 90.67 fs. This reference represents the intrinsic pulse duration of the laser without any dispersion or nonlinear effects and serves as the fundamental reference point for all subsequent pulse width comparisons. Compared with the temporal profile of a reference laser in air (90.67 fs, Figure 3b (i)), both cuvette (152.65 fs, Figure 3b (ii)) and water (197.54 fs, Figure 3b (iii)) result in an increase of pulse duration due to a positive chirp effect (d^2^ *n*/d*λ* ^2^ > 0). However, a significant pulse compression was observed in the presence of GO with a solution density of 1 mg/mL (138.66 fs, Figure 3b (iv)). In this case, it is noticeable that the two media of water and cuvette are involved. To observe the intrinsic effect of GO, a freestanding GO with the same density of 1 mg/mL was prepared. To hold such a fragile structure of freestanding GO, we made a 3D-printed gasket. As shown in Figure 3b (v), a pulse compression effect in freestanding GO was indeed observed, with the pulse width reduced to 83.01 fs, which is even shorter than the input pulse.

For an additional verification of its origin, we used a frequency-resolved optical gating (FROG) technique to measure the phase of the laser pulse as shown in Figure 3c. Instead of a spectrally integrated photodetector, we used a CCD camera attached to monochromator to measure the SHG spectrum for delay time, which is spectrogram. Given the SHG spectrogram of air (Figure 3d (i)) and freestanding GO film (Figure 3d (ii)), both the phase and intensity profile of laser pulse (Figure 3e) were extracted for delay time, respectively. Despite the small thickness (∼300 nm) of GO film, 9% of pulse duration shortening was observed with a significant phase change.

In Figure 3f, the spectrum of *n*(λ) and d^2^ *n*/d*λ* ^2^ in the GO film are compared, and we found that an inflection point d^2^ *n*/d*λ* ^2^ = 0 is present near 800 nm. In most of the optical medium, the GVD with a positive chirp (d^2^ *n*/d*λ* ^2^ > 0) is likely observed in a Ti:sapphire laser, resulting in a pulse duration broadening. However, the chirp effect can be suppressed near the wavelength of d^2^ *n*/d*λ* ^2^ = 0. For the GVD compensation of chirp, diffraction gratings and a pair of prisms are often used, but this method needs a large space for optical alignment. Alternatively, chirped mirrors can be used, where a stack of dielectric layers are coated for the chirp compensation. They are also limited by low reflectivity and narrow bandwidth. On the other hand, the freestanding GO film held by a gasket (Figure 3a) is remarkably convenient to use for the compression of a Ti:sapphire laser.

As we tuned the central wavelength of Ti:sapphire laser at 790 nm, where a small positive chirp effect (d^2^ *n*/d*λ* ^2^ > 0) is involved, the quadratic phase curve of a reference pulse (Figure 3e) was expected to steepen a little or remain constant after passing through freestanding GO film However, an opposite result was obtained, i.e. the phase curve became less steep through freestanding GO film, and the shortened pulse intensity also became asymmetric. These results suggest that a nonlinear optical effect is also involved in the pulse shortening, and SA is a possible origin.^19^ While an intense pulse travels through a saturable absorber, the leading edge of the pulse becomes attenuated more than the trailing part. As a result, asymmetric steepening of the temporal intensity profile can be induced.

### Nonlinear Optical Properties of Freestanding GO Films

As shown in Figure 4a, an open-aperture Z-scan was performed to investigate the NLO absorption in a freestanding GO film, where Ti:sapphire laser with 140 fs pulse duration was used. For a freestanding GO film located at the focal point (*z* = 0) (Figure 4a (i)), the transmitted laser intensity (I_T_) shows three different characteristic regions with increased input fluence (*I*_0_) (Figure 4b). Unless excitation is strong enough to cause NLO effects, *I*_T_ is proportional to *I*_0_ (*I*_T_ ∼ 0.26*I*_0_) with a constant slope of transmittance 0.26. However, as the incident laser fluence increases beyond 5 × 10^–1^ μJ cm^–2^, *I*_T_ deviates gradually from a linear dependence, and photobleaching becomes significant with increasing excitation up to a few μJ cm^–2^. This result can be attributed to SA. With further increased excitation (>2 μJ cm^–2^), a photoinduced absorption occurs, where the slope becomes less than the linear transmittance (d*I*_T_/d*I*_0_ < 0.26).

**Figure 4:**
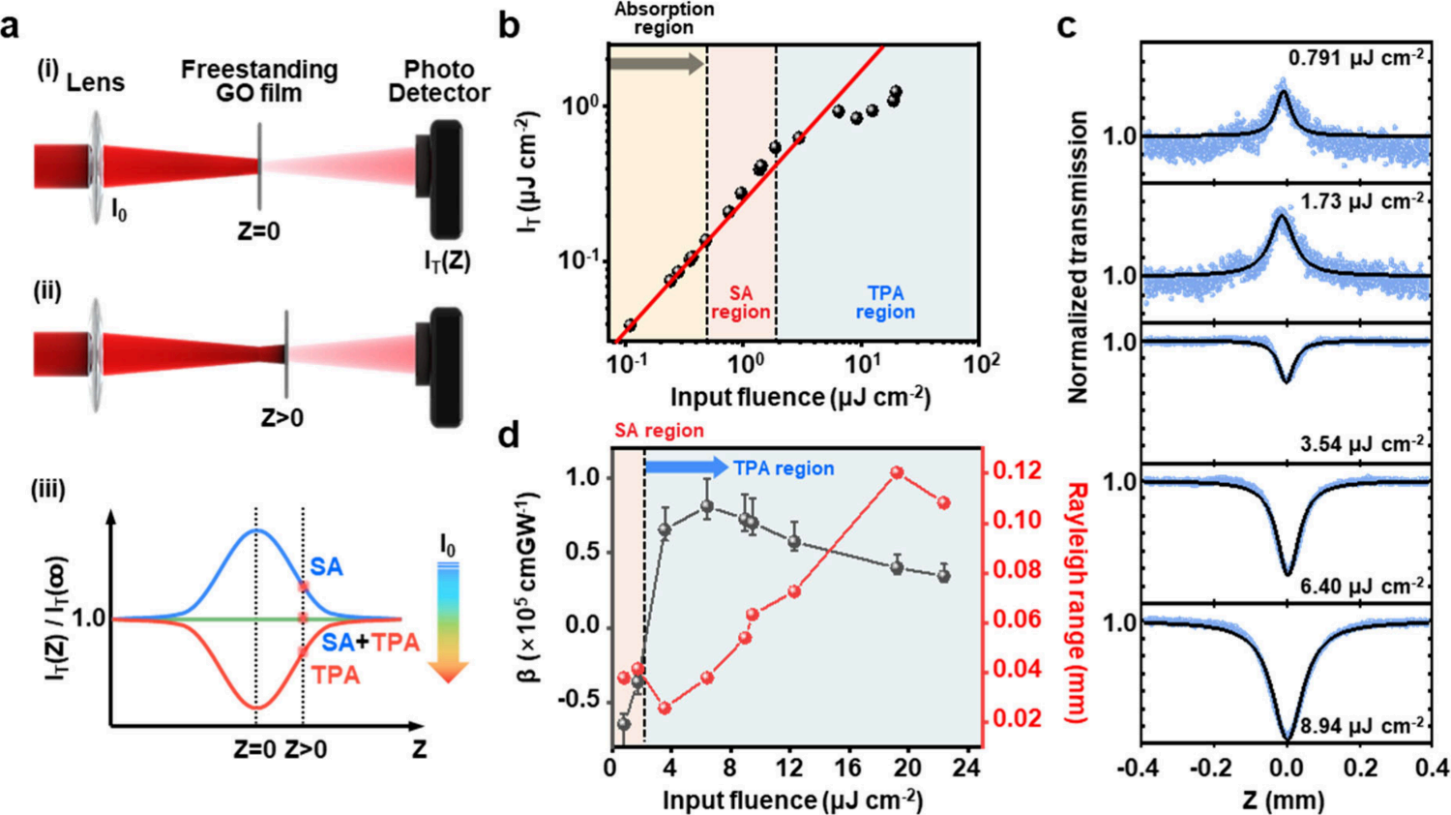
(a) The open aperture Z-scan method is shown schematically. (b) Provided that a free-standing GO film is located at the focal point (*z* = 0) as shown schematically in a-(i), the transmitted laser intensity (*I*_T_) shows three characteristic regions as the input fluence (*I*_0_) increases. (c) Z-scan results for various input fluences were obtained as shown schematically in a-(iii). (d) Input fluence dependence of the nonlinear absorption coefficient (β) and Rayleigh range were obtained by fitting with a theoretical model. The error bars in β reflect the uncertainty due to sample thickness variation across the film (+32 nm, −67 nm for the 293 nm film).

It was known that the *sp* ^2^ hybrid orbitals in GO comprise nanographene domains in the middle of the *s* *p* ^3^ matrix, where the nanographene domains are separated by boundaries comprising a network of epoxy and hydroxyl-bonded *s* *p* ^3^ orbitals of carbon atoms.^52^ Therefore, the heterogeneous NLO optical response can be tailored by the regulation of *sp* ^2^–*s* *p* ^3^ composite domains.^52,53^ According to time-dependent density functional theory calculations, an energy gap of ∼0.5 eV arises from the localized *sp* ^2^ nanographene domain with a diameter of ∼3 nm, which consists of ∼100 aromatic rings. If the size of a nanographene domain decreases below 20 aromatic rings, an increased energy gap of ∼2 eV can be obtained.^53^ On the other hand, *s* *p* ^3^ orbitals of carbon atoms bonded with oxygen-containing functional groups show a large energy gap ranging from 2.7 to 3.1 eV.^54^ With moderate excitation (∼10^–^^1^ μJ cm^–^^2^), the *s* *p* ^3^ matrix is still transparent to 800 nm Ti:sapphire laser light (∼1.55 eV), but the state filling effect is likely to occur in the confinement levels of the *sp* ^2^ nanographene domains. Consequently, the SA becomes dominant.^52^ With strong excitation (∼10 μJ cm^–^^2^), the large energy gap in the *s* *p* ^3^ matrix begins to allow a two-photon absorption (TPA) as well as an excited-state absorption (ESA).^52,53^ Although the *sp* ^2^ domains still cause simultaneous SA, the SA effect is overwhelmed by the photoinduced absorption of TPA and ESA in the *s* *p* ^3^ matrix.

For a quantitative analysis of the NLO absorption, the sample position is scanned around the focal position (*z* = 0) (Figure 4a (ii)), whereby excitation dependent *I*_T_ at the focal position is converted to a *z*-dependent *I*_T_ using the Gaussian beam focusing. Figure 4a (iii) shows schematically how *I*_T_(z) changes with increasing *I*_0_. Provided that SA appears with moderate *I*_0_, *I*_T_(*z*) near the focal point increases compared to the converging *I*_T_(∞) at large distances *z*. However, as the TPA overcomes the SA with strong excitation,*I*_T_(*z*) near the focal point decreases gradually. In Figure 4c, excitation input fluence dependence of the Z-scan *I*_T_(*z*) in free-standing GO film is shown. When fitted with a theoretical model for the open aperture Z-scan method,^55^ the NLO absorption coefficient (β) and Rayleigh range (*z*_0_) were estimated with increasing *I*_0_. While β determines the NLO absorption change Δ*α* = *βI*_0_, *z*_0_ represents the distance along the beam propagation direction from the waist (*z* = 0) to the place where the area of the cross section becomes doubled.

As shown in Figure 4d, the sign of β changes from negative to positive with increased input fluence due to a transition from the SA (β < 0) to the photoinduced absorption of TPA and ESA (β > 0).^52,53^ To account for experimental uncertainty, the error bars in β reflect the variation in film thickness measured across the sample, as shown in Figure 2f, where the average thickness is ∼293 nm. Compared to other work, our free-standing GO film gives rise to a photoinduced absorption at very weak excitation fluence (a few μJ cm^–2^), and its coefficient (β ∼ 10^4^ cm GW^–1^) is also huge. In GO solution dispersed in *N*, *N*-Dimethylformamide (DMF),^52,56^ a strong excitation fluence (∼10^8^ J cm^–2^) was necessary for TPA, where β ∼ 10^–2^ cm/GW was obtained using a femtosecond pulse at 800 nm. In GO films on a glass substrate, β was found to increase with decreasing *I*_0_ when compared with the results in a GO solution. For example, β ∼ 31 cm/GW was observed in an as-prepared GO film with ∼140 nm thickness^50^ at an excitation fluence of 13 mJ cm^–2^. With reduced thickness of GO films, β is enhanced up to ∼12 times (β∼10^2^ cm/GW). Provided a thick GO film was prepared (∼2 μm), β was found to increase up to 40000 cm GW^–1^ at 32 μJ cm^–2^.^19^ In the case of thick GO films, localized *sp* ^2^ domains increase with a smaller *sp* ^3^ matrix located mostly around the edges of the GO films. Our free-standing GO film provides a large NLO absorption coefficient up to β ∼ 80000 cm GW^–1^, but the thickness (∼300 nm) and the input fluence (a few μJ cm^–2^) are surprisingly small. Compared to reduced graphene oxide (rGO), which contains fewer oxygenated groups and a more continuous π-conjugated structure, our free-standing GO film exhibits stronger saturable absorption at low excitation fluence. This distinction arises from the discrete sp^2^ nanodomains within the sp3 matrix in GO, as opposed to the extended sp^2^ networks in rGO. As shown in previous studies,^19,21^ the β of rGO is significantly lower than that of GO under similar excitation conditions, underscoring the critical role of oxygen functionalities in enhancing the NLO response. Although further study is necessary to reveal the exact origin of the large NLO absorption, the density of GO and the matrix effects of solvent and polymers^57^ are possibly involved.

While the transition from saturable absorption to photoinduced absorption occurs in GO with increased *I*_0_, it was known that the refractive index decreases. Given *n*(*I*_0_) = *n*_0_ + *n*_2_ *I*_0_, the NLO term (*n*_2_ *I*_0_) decreases as the photoinduced absorption emerges^19^ while the linear refractive index *n*_0_ is constant. Because the Rayleigh length is also proportional to the nonlinear refractive index as *z*_0_∼*n*(*I*_0_), the abrupt decrease of *z*_0_ at ∼3.5 μJ cm^–2^ (Figure 4d) indicates the onset of photoinduced absorption. However, with increased *I*_0_ over 4 μJ cm^–2^, *z*_0_ extends gradually. This result is associated with laser-induced reduction of GO.^19,58^ As the linear refractive index *n*_0_ of reduced GO (∼3.06) is far larger than that of GO (∼2.27) at 800 nm, the increase of *n*_0_ by the laser-induced reduction process overwhelms the decrease of the NLO term *n*_2_ *I*_0_ for increasing *I*_0_. With the input fluence over ∼8 μJ cm^–2^, a gradual decrease of β was observed. Due to the absence of a substrate, a free-standing GO film is less efficient in heat dissipation. Therefore, thermal degradation effects are a possible origin. Nevertheless, a free-standing GO film is quite convenient to use unless the laser fluence is strong enough to cause thermal damage. Technically this problem can possibly be resolved using efficient thermal conducting materials for the GO holder. In this case, the heat dissipation occurs through the lateral plane. It is also noticeable that a pulse compression can be achieved due to the strong SA effect even if an unfocused laser beam with 2 mm diameter is transmitted through free-standing GO film. Beyond this practical robustness, the advantages of graphene oxide for femtosecond pulse compression extend further. Its broadband nonlinear optical response, facile chemical tunability, and compatibility with solution-based, scalable fabrication render GO highly versatile. Without the need for conventional bulk components that require complex alignment setups or multilayer dielectric stacks, GO facilitates the implementation of ultrathin, free-standing film-based compressors with tunable thickness and excellent mechanical flexibility. These features establish it as a convenient and compact optical filter for ultrafast pulse shaping in next-generation photonic platforms.

In the broader context of pulse compression technologies, it is also important to position this work relative to existing commercial systems. Commercial compressors, such as hollow fiber compressors and grating or prism-based systems, achieve high compression ratio through complex dispersion management and require bulky optics and precise alignments. In contrast, our freestanding GO film presents a compact, all-passive approach to pulse compression via saturable absorption without active dispersion control. This intrinsic material property manifests compression at moderate excitation fluences (∼1 μJ cm^–2^) in an ultrathin (∼300 nm) film format, highlighting potential for integration into compact, alignment-free ultrafast photonic devices. Although direct benchmarking of the pulse compression ratio and physical footprint against commercial systems is beyond the scope of this material-focused study, future research will aim to incorporate freestanding GO films into complete laser oscillator setups to enable quantitative performance comparison. Furthermore, our novel fabrication approach based on thermocapillary-assisted evaporative self-assembly using perforate templates offers scalable production of these freestanding GO films, presenting a promising alternative to conventional chirped mirrors or bulk optics compressors. This expands the applicability of GO saturable absorbers beyond fiber laser configurations^59,60^ to free-space ultrafast photonics, an area relatively unexplored to date.

## Conclusion

Employing the self-assembly of GO sheets into a stacked configuration by spontaneous solvent evaporation, we have developed a simple approach to produce a freestanding GO. Surprisingly we found that the freestanding GO film exhibits a significant pulse compression effect on Ti:sapphire femtosecond laser pulses, where SA from the sp^2^ nanographene domains is the main origin, but a suppressed chirp effect is also associated due to the presence of an inflection point in the refractive index spectrum (d^2^ *n*/d*λ* ^2^ ≈ 0) near 800 nm. Compared to other work on GO, the transition from SA to TPA is induced at low excitation fluence (a few μJ cm^–2^), and the NLO absorption coefficient also increases up to β∼80000 cm/GW. Because of the enhanced NLO absorption, pulse compression in a freestanding GO film can be achieved easily via SA even for unfocused Ti:sapphire femtosecond pulses at low excitation fluence (∼10^–1^ μJ cm^–2^). While the current pulse compression techniques are based on the chirp compensation by stacked layers or complicate alignments, the pulse compression of free-standing GO is only based on novel linear and nonlinear optical properties. Therefore, a free-standing GO film can be a promising alternative as an ultrafast pulse compressor, which is compact and convenient to use.

## Supplementary Material



## Data Availability

The data that support these findings are available from the corresponding author upon request. Source data of all figures are provided with this paper.

## Notes

Synthesis and characterization of freestanding GO membranes (Figures S1–S7), nonlinear optics experimental setup (Figure S8), estimation and calibration of refractive index spectrum using Kramers–Kronig and Fresnel analysis (Figures S9–S10), group refractive index measurement using intensity cross-correlation (Figure S11), laser-induced damage threshold under CW excitation (Figure S12), chirped pulse simulation in dispersive GO medium (Figures S13–S14) (PDF)
