# Supplementary material for: Giant Nonlinear Optical Absorption of Freestanding Graphene Oxide Films for Femtosecond Pulse Compression
Source: ACS Appl Mater Interfaces. 2025 Jul 18;17(30):43476–87. doi: 10.1021/acsami.5c10300 (PMC12314857; doi:10.1021/acsami.5c10300)
Supplement: Supplementary file 1 [file am5c10300_si_001.pdf]

Supporting information

Giant Nonlinear Optical Absorption of Freestanding Graphene Oxide Films for Femtosecond Pulse Compression

Rowoon Park [ab†], *Sang-Hyuk Park* [c†], *Minwoo Kim* [a†], *Minju Kim* [a], *Seungho Park* [a], *Young Woo Kwon* [b], *Songyi Lee* [d], *Kwangseuk Kyhm* [a*], *Suck Won Hong* [ab*], *and Robert A. Taylor* [c*]

[a]Department of Optics and Mechatronics Engineering, Department of Cogno-Mechatronics Engineering, and College of Nanoscience and Nanotechnology, Pusan National University, Busan 46241, Republic of Korea

[b]Engineering Research Center for Color-Modulated Extra-Sensory Perception Technology, Pusan National University, Busan 46241, Republic of Korea

[c]Department of Physics, University of Oxford, Oxford, OX1 3PU, UK

[d]D Department of Chemistry, Pukyong National University, Busan 48513, Republic of Korea

[†]These authors contributed equally to this work.
*E-mail: kskyhm@pusan.ac.kr(K.K.);swhong@pusan.ac.kr(S.W.H.);
robert.Taylor@physics.ox.ac.uk (R.T)

A. Supplementary note

Supplementary Note 1. Estimation of refractive index spectrum.

In Figure S9a, the absorption coefficient spectrum $\alpha(\lambda)$ of GO solution was obtained, which is associated with the extinction coefficient spectrum as $\alpha(\lambda) = \frac{2\pi}{\lambda}\kappa(\lambda)$. According to the Kramers-Kronig relations[1], the real and imaginary spectrum of complex refractive index are associated[2,3]. Given the imaginary spectrum of extinction coefficient $\kappa(\lambda)$, the real spectrum of refractive index can be obtained by $n(\omega) = 1 + \frac{1}{\pi}\int_{-\infty}^{\infty} \frac{\omega'\kappa(\omega')}{\omega^2-\omega'^2} d\omega'$, where $\omega = \frac{2\pi c}{\lambda}$. In Figure S9b, a spectrum $f_n(\lambda)$ corresponds to a converted real part from the imaginary spectrum. However, it is noticeable that the practical integration range is finite. As a result, the magnitude of $f_n(\lambda)$ still needs calibration although the spectrum is similar to that of the real refractive index. Figure S9c shows the refractive index spectrum calibrated from $f_n(\lambda)$, where the two refractive indices at 404 nm and 633 nm were used. For precise refractive index measurement, the Fresnel reflection for the two perpendicular linear polarizations was utilized[4].

As shown schematically in Figure S10a, transverse electric (TE) and transverse magnetic (TM) polarized light can be selected for incident and reflected light[5]. Regarding the ratio of reflected intensity to incident intensity for the two perpendicular polarized lights, TE-reflectance (R_{TE}) and TM-reflectance (R_{TM}) can be obtained for increasing incident angle (θ), respectively. In Figure S10b and S10c, incident angle dependence of R_{TE}/R_{TM} are shown for 404 nm and 633 nm, respectively. Given the absorption coefficients obtained in Figure S9a, the incident angle dependence of R_{TE}/R_{TM} were also compared with the Fresnel equations[1], where the gradual increase and the maxim range near the Brewster angle were fitted by optimizing the refractive index as a fitting parameter. When incident angle is large ($\theta > 75^\circ$), the angle dependence deteriorates due to scattering.

Supplementary Note 2. Group refractive index measurement.

Group refractive index of medium was measured by intensity cross-correlator shown in Figure S11a, where one of the split laser pulses pass through solution sample in cuvette, resulting in a time delay compared with the pulses in the other path. The delayed pulse is overlapped with a reference pulse in nonlinear crystal (BBO) to produce second harmonic generation, and the intensity correlation for delay time can be considered temporal intensity pulse profile. As shown schematically in Figure S11b, time delay induced by quartz cuvette

and solution was compared with reference pulse propagating in air, respectively. Figure S11c shows the temporal intensity profiles of various samples compared with that of reference in air. Compared with the reference pulse in air, the group refractive index n_g of medium results in a relative time delay $\Delta t = \frac{2n_g d}{c}$, where d is the thickness of medium and c is the speed of light[3]. Regarding the double transmission along the sample path in Figure S11a, the total optical length is $2n_g d$. The quartz medium of empty cuvette causes a time delay of Δt ~7617 fs. As the quartz becomes filled with water, an increased delay of Δt ~30435 fs was observed. With an additional GO of 1 mg mL^{-1}, Δt~30453 fs was observed. Consequently, the group refractive index n_g of medium can be obtained with the cuvette size. Given the refractive index spectrum $n(\lambda)$, the measured group refractive index at the central wavelength (λ_0) can be verified with $n_g(\lambda_0) = n(\lambda_0) - \lambda_0 \frac{dn}{d\lambda_0}$.

Supplementary Note 3. Laser-induced damage in the freestanding GO film.

With intense light excitation over a threshold, GO shows a phase transition to reduced GO. Furthermore, the laser-induced heat in a freestanding structure is unlikely dissipated due to the absence of substrate, and this may cause a permanent damage. To investigate the laser-induced damage, freestanding GO film was exposed to continuous-wave laser with 730 nm wavelength and 2 mm spot size, and the transmitted light power was measured for increasing excitation power, as shown in Figure S12a. The output power linearly increases up to 20 mW of excitation power, but gradually deviates from the linear dependence for increased excitation power (> 20 mW). With excitation power above ~ 40 meV, the output power a significant decrease for excitation power. In this case, the degraded area is not recovered, and the damage spot shows an absorption increase. In Figure S12b, optical microscope image shows the damaged dark spots in a free-standing GO film.

Supplementary Note 4. Calculation of chirped pulse.

When laser pulse propagates in a dispersive medium with $n(f)$, the pulse duration changes due to the chirp, which is determined by the second-order phase term in time domain. In spectrum domain (f), the chirp is associated with the group velocity dispersion or group delay dispersion (GDD) in the second-order phase term[6]. In Figure S13a and S13b, we have calculated the temporal pulse profiles in three examples of positive and negative chirped and unchirped cases, whereby the validity of our model can be confirmed. In Figure S13a, a Gaussian laser spectrum is given with the three cases of optical dispersion with $n(f)$ (solid line) and $\frac{dn^2}{df^2}$(dotted line). In Figure S13b, the temporal change of laser pulse profile can be seen for the three cases. Given an initial unchirped pulse, the positive chirped medium ($\frac{dn^2}{df^2} > 0$) gives rise to an increase of pulse duration during propagation. It is noticeable that the negative chirped medium ($\frac{dn^2}{df^2} > 0$) also causes an increase of pulse duration compared with an unchirped initial pulse. In a practical point of view, positive chirped pulses are likely generated from the laser cavity. In this case, a negative chirp system can shorten the pulse duration. Nevertheless, most of optical media are of positive chirp.

In Figure S14a, the chirp effect was considered at three central laser wavelengths (707 nm, 802 nm, and 926 nm) regarding the measured spectrum of $n(\lambda)$ and $\frac{d^2\mathrm{n}(\lambda)}{\mathrm{d}\lambda^2}$ in GO, where

positive chirp $\frac{d^2\mathrm{n}(\lambda)}{\mathrm{d}\lambda^2} > 0$ and negative chirp $\frac{d^2\mathrm{n}(\lambda)}{\mathrm{d}\lambda^2}$<0 are expected below and above the critical wavelength 802 nm pulse. In Figure S14b, the propagation thickness dependence of pulse duration was compared for the three different central wavelengths. Because the initial pulse was assumed to be unchirped, all the pulse duration becomes increased for propagation thickness. Even if the central laser wavelength is tuned at the critical wavelength 802 nm, chirp effect occurs within the laser linewidth. Figure S14c, d, and e show how the initial pulse profile with different central wavelength changes as it propagates through GO film. This theoretical calculation suggests that the chirp effect is not a main origin of the pulse shortening in GO, and nonlinear effect should be involved. Therefore, this is also consistent with our conclusion such that saturable absorption plays a dominant role for the pulse shortening in GO film.

B. Supplementary figures

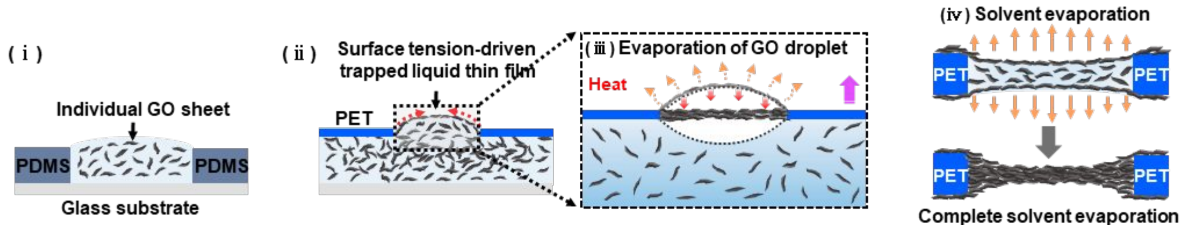

Figure S1. The detailed side view for forming the freestanding GO membrane bordering on PET edge.

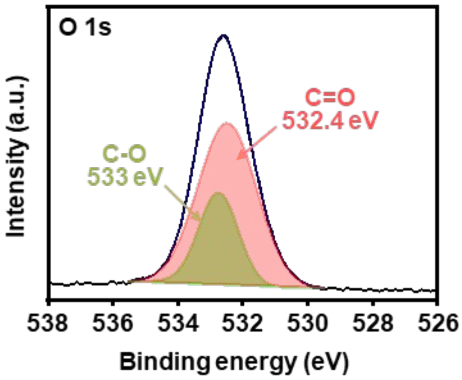

Figure S2. The O1s XPS spectra of the freestanding GO thin films.

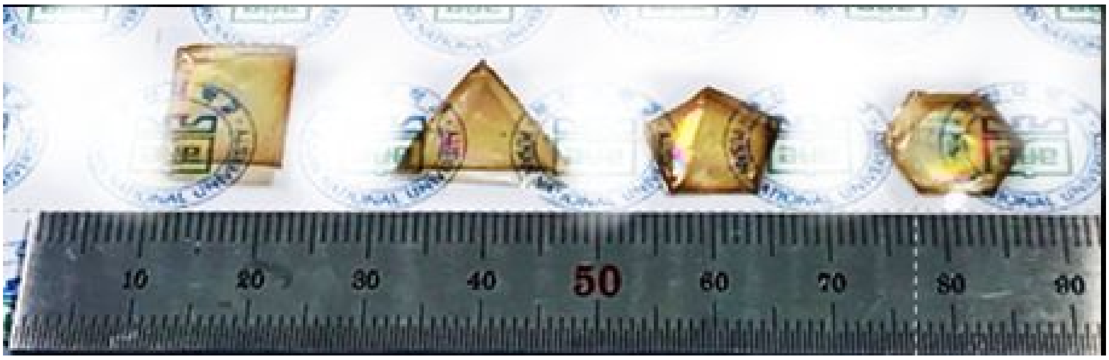

Figure S3. Various types of large-scale freestanding GO membrane specifically shaped by the perforated PET guide films.

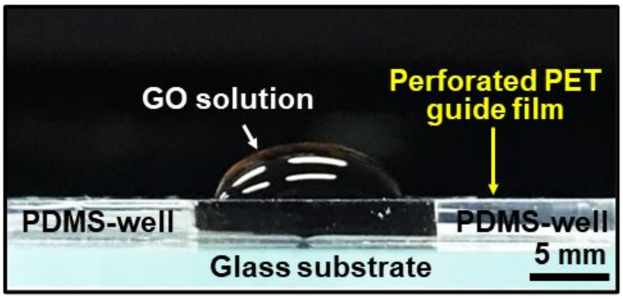

Figure S4. Digital image showing the fabrication process of a freestanding GO thin films. A GO droplet is entrapped in a confined geometry consisting of a PDMS well and a perforated PET guide film while maintaining a contact angle of 70°.

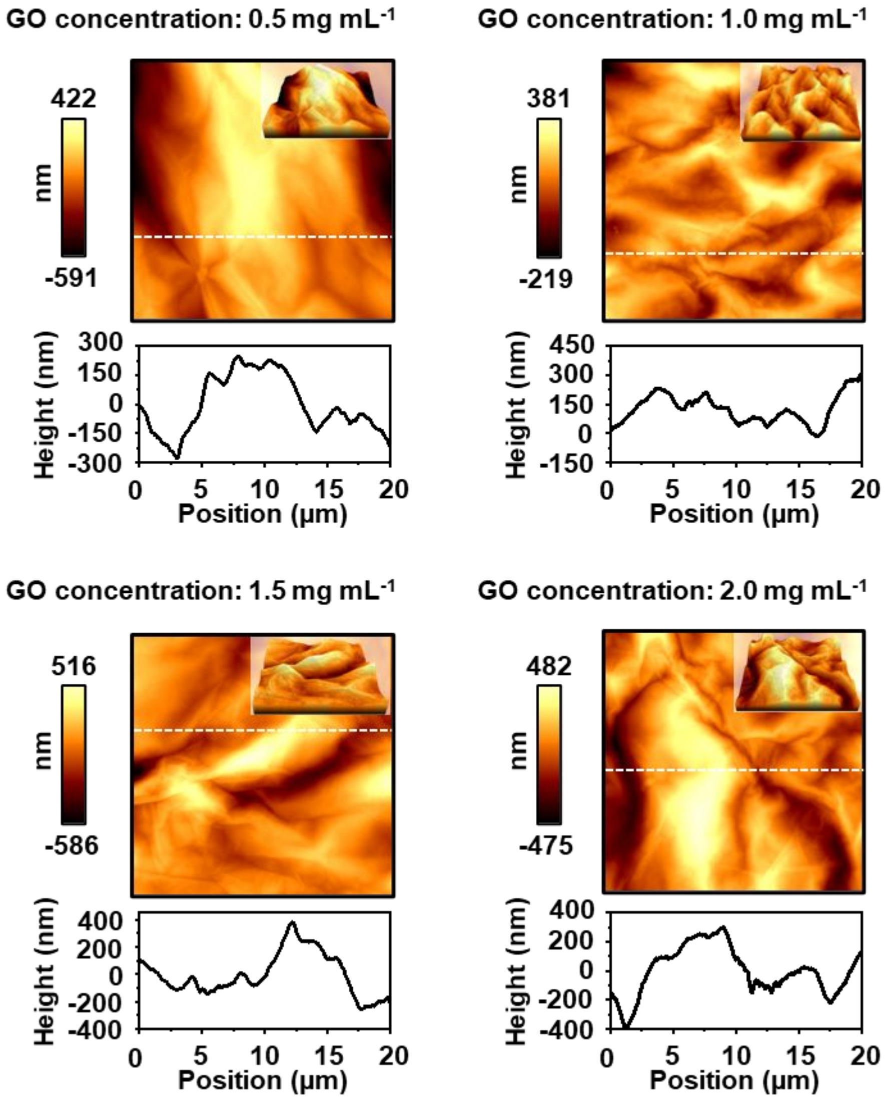

Figure S5. AFM images and height profiles for freestanding GO thin films prepared using GO concentration at 0.5, 1.0, 1.5, and 2.0 mg mL^{-1}

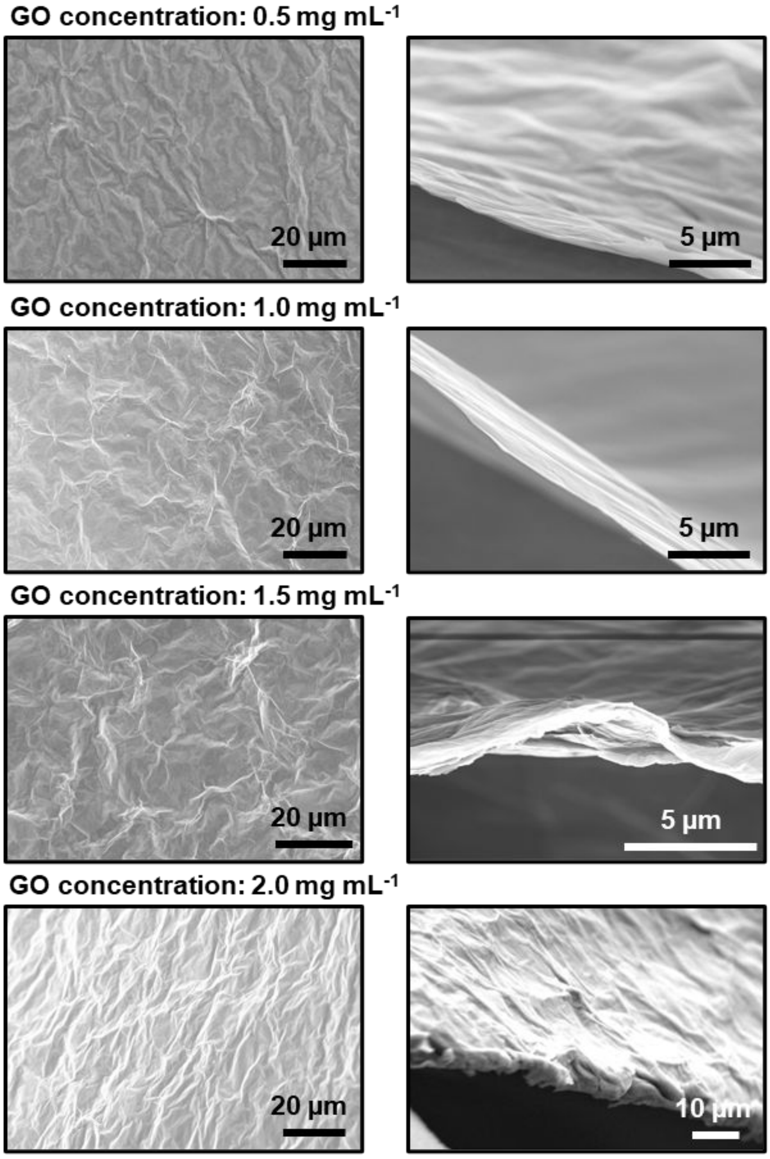

Figure S6. The SEM image displaying the surface morphology of freestanding GO thin film fabricated using GO concentration at 0.5, 1.0, 1.5, and 2.0 mg mL^{-1}; as the GO concentration increases, the film thickness tends to increase.

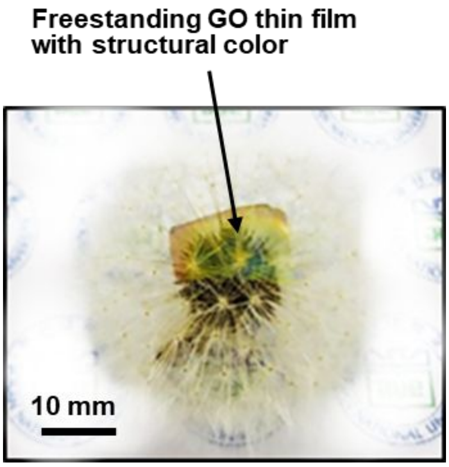

Figure S7. Top-view image of the freestanding GO thin films placed on the dandelion seed.

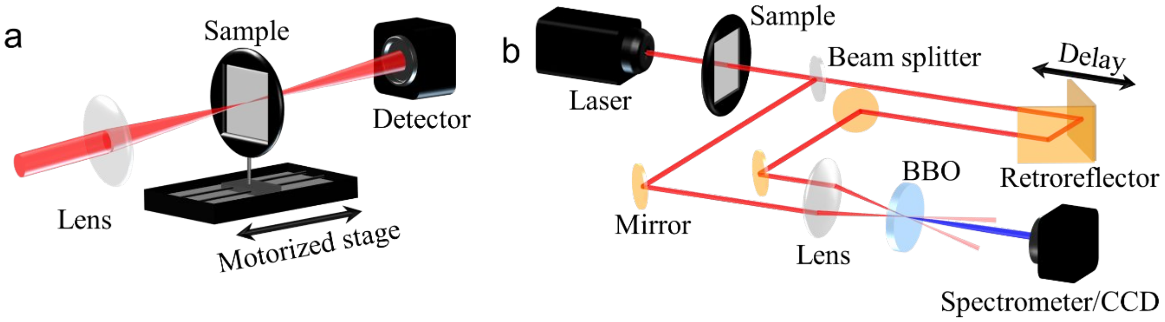

Figure S8. a) Z-scan setup for nonlinear absorption coefficient. b) Auto-correlation setup for frequency-resolved optical gating (FROG), where the frequency doubled second harmonic generation (SHG) spectrum is measured for time delay.

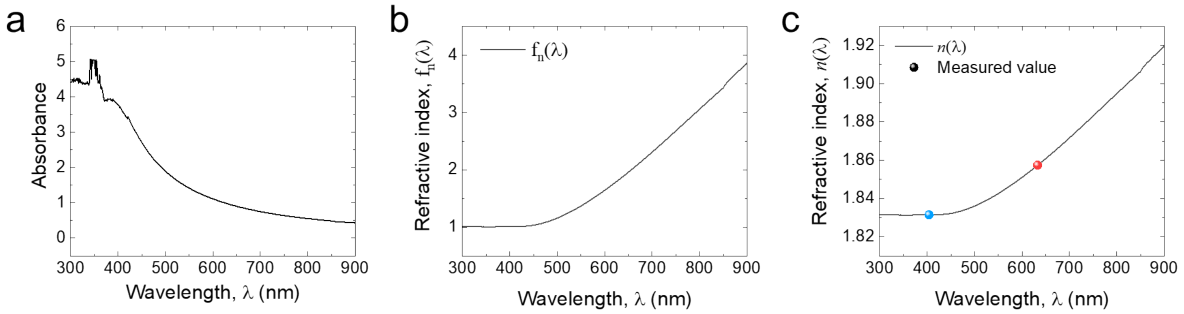

Figure S9. a) Given the absorbance spectrum of GO solution (1mg/mL), b) A spectrum $f_n(\lambda)$ can be calculated using the Kramers-Kronig relations, which corresponds to the converted spectrum. c) With the two refractive indices measured at 404 nm and 633 nm, a calibrated refractive index spectrum can be obtained from $f_n(\lambda)$.

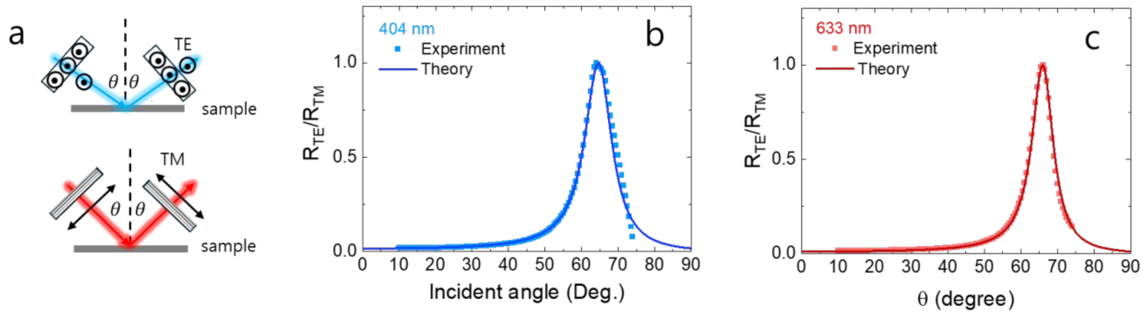

Figure S10. a) Schematics shows incident angle (θ) dependence of reflectance for TE and TM polarized light, where two different colors of light (404 nm and 633 nm) were used. To estimate refractive index, the ratio of TE-polarized reflectance to TM-polarized reflectance for incident angle were fitted by the Fresnel equation at 404 nm (b) and 633 nm (c), respectively.

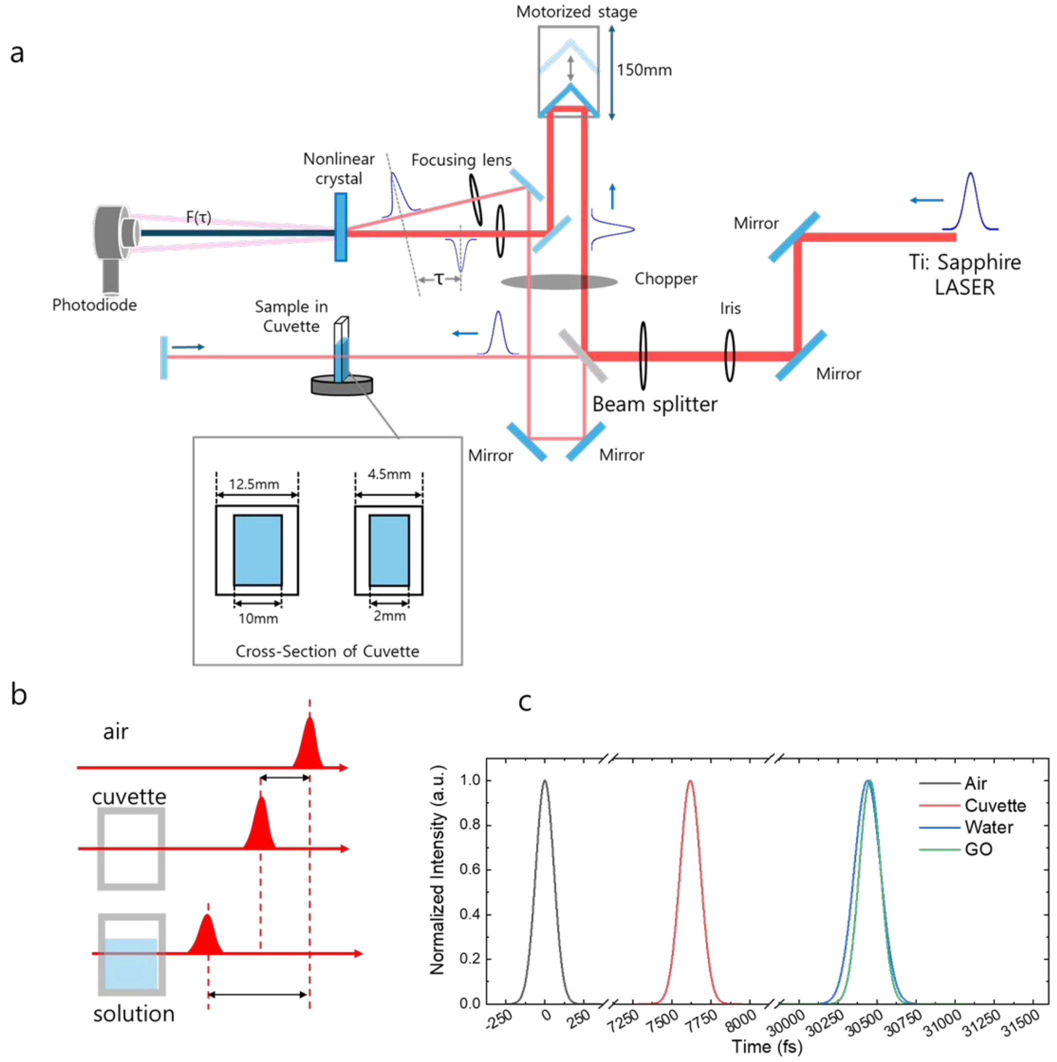

Figure S11. a) Group refractive index measurement setup up, where solution sample is contained in a cuvette. b) Schematics shows that pulse propagation become delayed due to the group refractive index of medium compared to the refence case in air. c) Temporal delay time of pulse in air, empty cuvette, cuvette filled with water and GO solution (1mg/1ml) are compared.

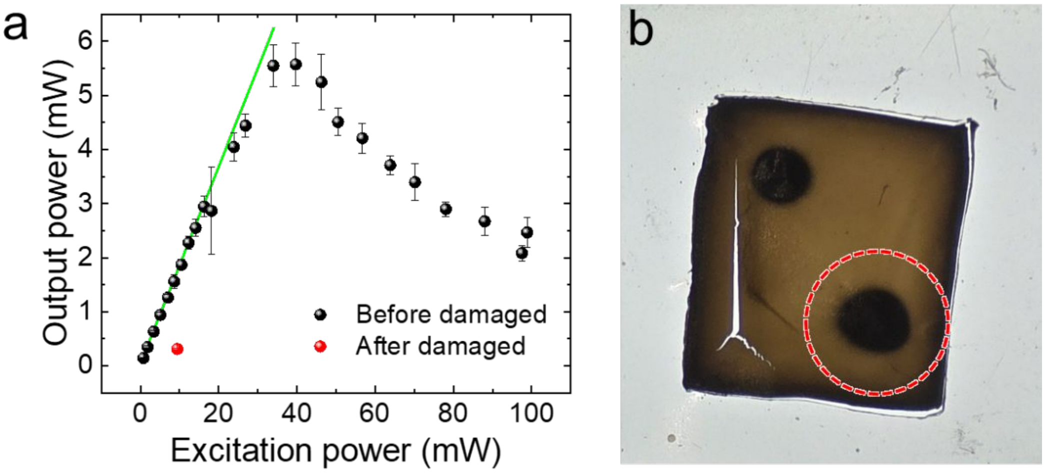

Figure S12. a) Transmitted output power in freestanding GO film for increasing excitation power, where the linear green line is for guidance. b) Optical microscope image shows damaged spots. The freestanding GO film is likely torn while detached from the holder.

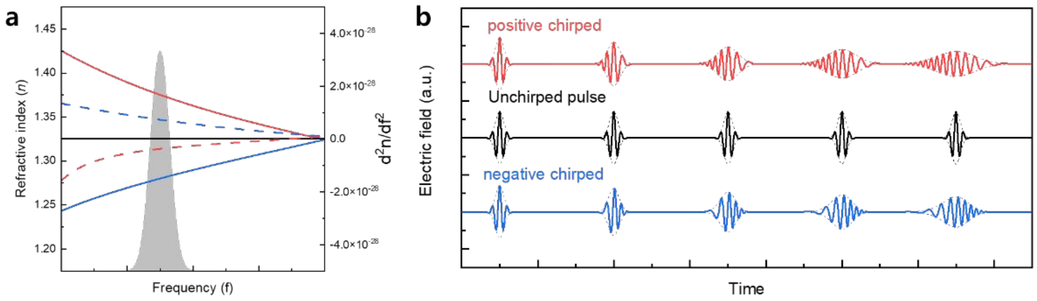

Figure S13. a) Given three theoretical spectra of linear refractive index $n(f)$ (solid line) and its second-order derivative $\frac{dn^2}{df^2}$(dotted line), negative (blue) and positive (red) chirp effects of Gaussian pulse were considered in comparison to non-dispersive unchirped case (black). b) The duration of pulses passing through unchirped (black) medium is unchanged, while pulses propagating in negatively (red) and positively (blue) chirped media show an increase in pulse duration.

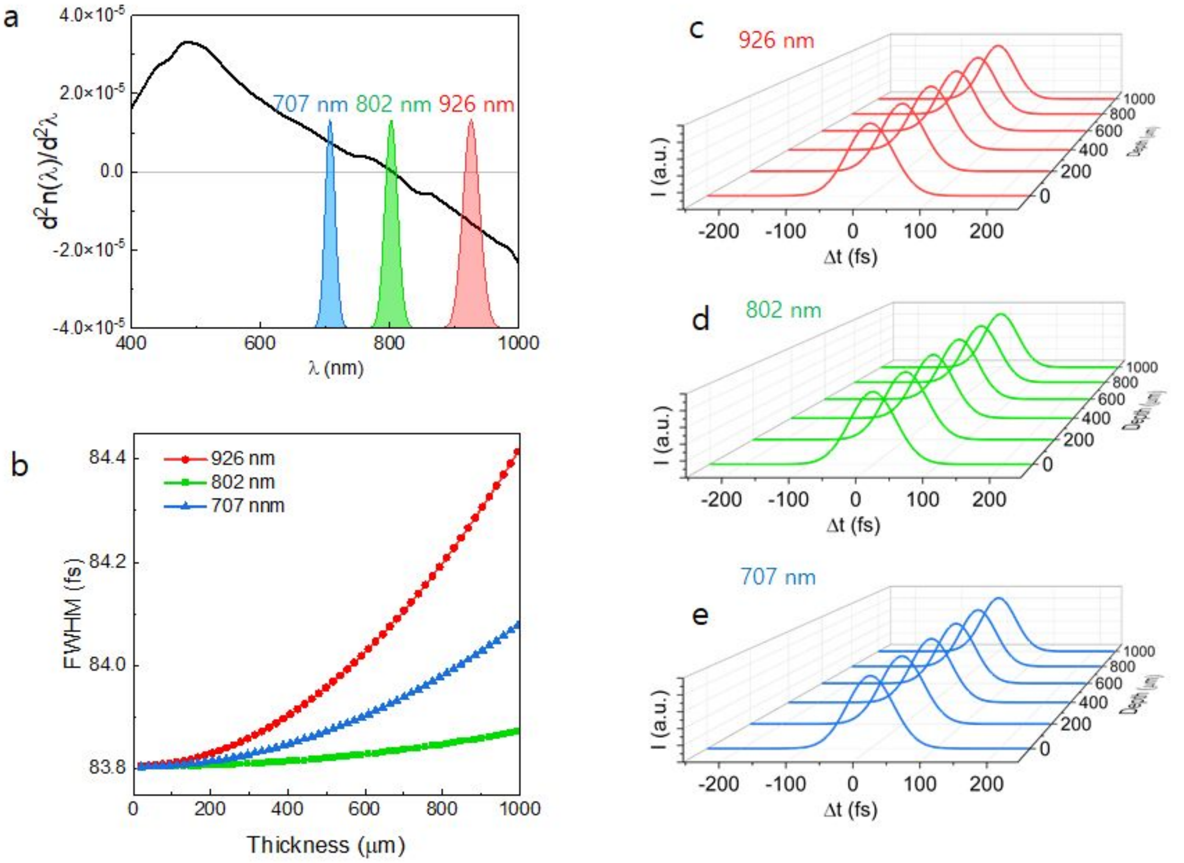

Figure S14. a) Three different wavelengths of femtosecond laser pulse are considered with the spectrum of second-order wavelength derivative to refractive index $\frac{d^2}{d\lambda^2}n(\lambda)$ in a dispersive GO film. b) For increasing propagation sample thickness, the pulse duration increase (FWHM) was calculated for three central wavelengths (707 nm, 802 nm, and 926 nm). c,d,e) As propagation depth increases, gradual increase of temporal pulse profile was calculated for the three wavelengths.

C. Reference

(1) Valerio Lucarini , K.-E. P., Jarkko J. Saarinen , Erik M. Vartiainen. *Kramers-Kronig Relations in Optical Materials Research*; Springer Berlin, Heidelberg, 2005.

(2) Kazemi, M. A.; Elliott, J. A. W.; Nobes, D. S. The influence of container geometry and thermal conductivity on evaporation of water at low pressures. *Sci Rep* 2018, ***8*** (1), 15121.

(3) Bor, Z.; Osvay, K.; 1, B. R.; Szabó, G. Group refractive index measurement by Michelson interferometer. *Optics Communications* 1990 ***78***, 109-112.

(4) Hecht, E. *Optics*; Addison-Wesley, 2005.

(5) Tompkins, H. G.; Irene, E. A. *Handbook of ellipsometry*; William Andrew Pub. ; Springer, 2005.

(6) Nussenzveig, H. M. *Causality and dispersion relations*; Academic Press, 1972.
